# Bifunctional quorum-quenching and antibiotic-acylase MacQ forms a 170-kDa capsule-shaped molecule containing spacer polypeptides

**DOI:** 10.1038/s41598-017-09399-4

**Published:** 2017-08-21

**Authors:** Yoshiaki Yasutake, Hiroyuki Kusada, Teppei Ebuchi, Satoshi Hanada, Yoichi Kamagata, Tomohiro Tamura, Nobutada Kimura

**Affiliations:** 1 Bioproduction Research Institute, National Institute of Advanced Industrial Science and Technology (AIST), 2-17-2-1 Tsukisamu-Higashi, Toyohira, Sapporo, 062-8517 Japan; 2 Bioproduction Research Institute, National Institute of Advanced Industrial Science and Technology (AIST), 1-1-1 Higashi, Tsukuba, Ibaraki, 305-8566 Japan; 3 Graduate School of Life and Environmental Sciences, University of Tsukuba, 1-1-1 Ten-nodai, Tsukuba, Ibaraki, 305-8572 Japan

## Abstract

Understanding the molecular mechanisms of bacterial antibiotic resistance will help prepare against further emergence of multi-drug resistant strains. MacQ is an enzyme responsible for the multi-drug resistance of *Acidovorax* sp. strain MR-S7. MacQ has acylase activity against both *N*-acylhomoserine lactones (AHLs), a class of signalling compounds involved in quorum sensing, and β-lactam antibiotics. Thus, MacQ is crucial as a quencher of quorum sensing as well as in conferring antibiotic resistance in *Acidovorax*. Here, we report the X-ray structures of MacQ in ligand-free and reaction product complexes. MacQ forms a 170-kDa capsule-shaped molecule via face-to-face interaction with two heterodimers consisting of an α-chain and a β-chain, generated by the self-cleaving activity of a precursor polypeptide. The electron density of the spacer polypeptide in the hollow of the molecule revealed the close orientation of the peptide-bond atoms of Val20SP-Gly21SP to the active-site, implying a role of the residues in substrate binding. In mutational analyses, uncleaved MacQ retained degradation activity against both AHLs and penicillin G. These results provide novel insights into the mechanism of self-cleaving maturation and enzymatic function of N-terminal nucleophile hydrolases.

## Introduction

*Acidovorax* sp. strain MR-S7 is a gram-negative bacterium that was isolated from activated sludge in a treatment system for penicillin G-polluted wastewater^1–3^. We previously reported that *Acidovorax* sp. strain MR-S7 is resistant to various β-lactam antibiotics and is able to degrade a broad range of *N*-acylhomoserine lactones (AHLs)^3^. AHLs are a class of signalling compounds involved in the bacterial intercellular communication known as quorum sensing^4^. Quorum sensing regulates many bacterial behaviours, including luminescence^5^, biofilm formation^2, 6–11^, signal turnover^12^, pigment production^13, 14^, antibiotic production^14, 15^, swarming^16^, and virulence^17–21^. AHL-degrading enzymes interfere with the AHL-mediated quorum sensing system in a process termed quorum quenching^22^. Different pathogens that are serious health threats, particularly in nosocomial infections, produce virulence factors under the control of a quorum-sensing system. Thus, quorum quenching could be a key strategy to interfere with bacterial infectious diseases^23–26^. AHL-acylase is one of the enzymes capable of inactivating AHLs via hydrolysis of the amide bond of the acyl side-chain to produce homoserine lactone (HSL) and fatty acid^3, 22^. Several enzymes exhibiting AHL-acylase activity have been reported. They include AiiD from *Ralstonia* sp. XJ12B^27^, PvdQ and QuiP from *Pseudomonas aeruginosa* PAO1^28–30^, AhlM from *Streptomyces* sp. M664^31^, AiiC from *Anabaena* sp. PCC7120^32^, Aac from *Shewanella* sp. MIB015^33^, and HacA and HacB from *P. syringae* B728a^34^.

**Figure 1:**
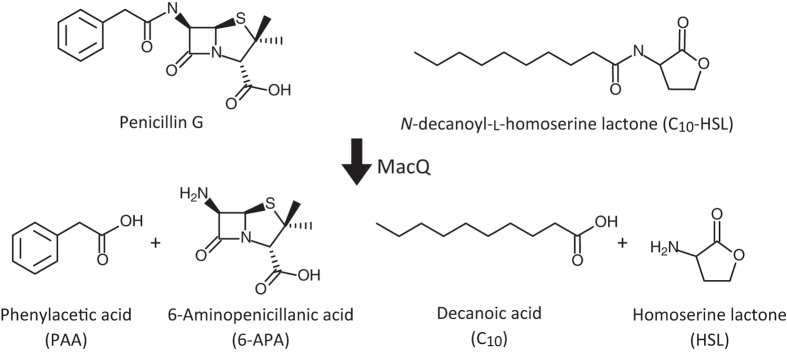
Amidohydrolysis of AHL (C_10_-HSL) and penicillin G catalyzed by MacQ. As shown in Table 1, MacQ is capable of degrading wide varieties of AHLs as well as penicillin G.

**Figure 2:**
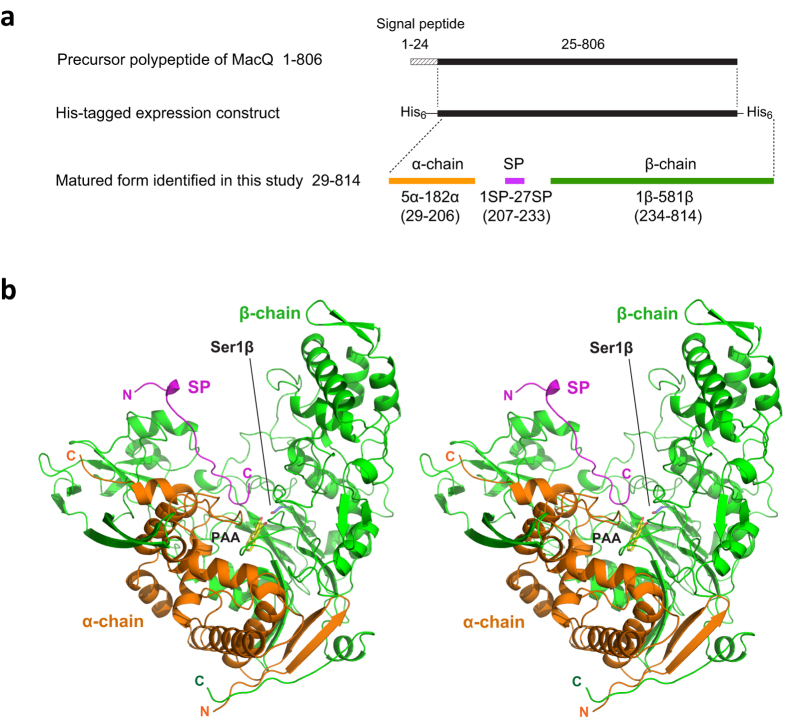
Heterodimeric structure of MacQ. (**a**) Schematic representation of the single MacQ precursor polypeptide and its self-cleaving maturation products. The N-terminal sequencing and MALDI-TOF/MS analysis indicated that the α-chain, SP, and β-chain correspond to residues 5α-182α (29–206), 1SP-27SP (207–233), and 1β-581β (234–814), respectively. The parentheses refer to the residue number of precursor polypeptide of MacQ including signal peptide sequence. The signal peptide (residue 1–24) was predicted with the SignalP 4.1 program (http://www.cbs.dtu.dk/services/SignalP/). (**b**) Stereo-view ribbon diagram of MacQ heterodimer with the SP generated by self-cleaving activity of a MacQ precursor single polypeptide chain. The α-chain, SP, and β-chain are coloured in orange, magenta, and green, respectively. The bound PAA and the catalytic Ser1β are also shown as stick models in yellow and blue, respectively, and are labelled.

We recently identified a gene (*macQ*) responsible for β-lactam antibiotic resistance of *Acidovorax* sp. strain MR-S7. The recombinant MacQ protein exhibited bifunctional acylase activity against various ranges of AHLs and against multiple β-lactam antibiotics, including penicillin G, ampicillin, and amoxicillin (Fig. 1)^3^. The MacQ protein is comprised of 806 amino acid residues, including an N-terminal signal peptide (Fig. 2a), and belongs to the N-terminal nucleophile (Ntn) hydrolase superfamily. Ntn hydrolases are synthesized as inactive precursor single polypeptides, which become activated by self-cleaving activity, generating the mature hetero-multimeric enzyme composed of α- and β-chains^35^. The penicillin G/cephalosporin*/*AHL-acylases are more homologous to MacQ among the Ntn hydrolases. The precursor polypeptide of these homologues harbours two distinct cleavage sites that enable the formation of a mature heterodimer composed of α- and β-chains, with the release of a spacer polypeptide (SP)^30, 36–39^. The N-terminal Ser/Thr/Cys of the β-chain is the catalytically essential residue, which acts as a nucleophile to initiate amide bond cleavage of various substrate compounds^40–43^. Extensive functional and structural analyses have been reported for Ntn hydrolase enzymes. Two enzymes, AhlM and KcPGA, exhibit acylase activity against both AHLs and penicillin G^31, 44^, similar to MacQ. However, the structures and substrate adaptation mechanisms of those bifunctional acylases are unknown.

Here, we describe the X-ray crystal structures of the bifunctional quorum-quenching and β-lactam antibiotic-degrading Ntn hydrolase MacQ in ligand-free form and in complex with reaction products, fatty acid, or phenyl acetic acid. The structures revealed that the three distinct polypeptide chains generated by self-cleaving activity assemble into a capsule-like higher oligomeric complex.

## Results and Discussion

### Recombinant expression of MacQ by *Escherichia coli*

The expression of *macQ* isolated from *Acidovorax* sp. strain MR-S7 was performed using *Escherichia coli* cells, as previously described^3^. The precursors of AHL-acylases and related enzymes are cleaved into three polypeptide chains (α-chain, internal SP, and β-chain) and the catalytically active mature enzymes are generated as a heterodimer consisting of α- and β-chains, with the SP being released^30^. As expected, SDS-PAGE analysis confirmed two bands at approximately 20 kDa and 62 kDa, which corresponded to the α- and β-chains of MacQ, respectively (Fig. 2a). Although we created an expression construct to produce recombinant MacQ with His_6_-tags at both the N- and C-termini (Fig. 2a), N-terminal sequence analysis for the 20 kDa band revealed that the origin of the α-chain begins at Ser29 and a truncated N-terminal His_6_-tag. MALDI-TOF/MS analysis predicted a subunit weight for the α-chain of 19.23 kDa. On the basis of these data, we presume that the α-chain of the purified sample consists of 178 amino acid residues from Ser29 (Ser5α) to Ala206 (Ala182α), with a theoretical molecular weight of 19.21 kDa (Fig. 2a). The cleavage site at Ala206 agreed well with that of the AHL-acylase homologue PvdQ from *P. aeruginosa* strain PAO1, as previously reported^30^. MALDI TOF/MS analysis also detected minor peaks between 19.2–19.6 kDa, suggesting that the cleavage position between the α-chain and SP might not be specific. The other cleavage site was at Ser234, which defines the Ntn residue of the β-chain (Ser1β).

### MacQ forms a capsule-shaped dimer of heterodimer containing SP chains

The crystal structures of MacQ, both ligand-free and in complex with the reaction products of decanoic acid (C_10_) and phenyl acetic acid (PAA), were determined at 2.6 Å, 2.2 Å, and 1.75 Å resolution, respectively. The initial atomic model of ligand-free MacQ was built by the molecular replacement method using the PvdQ heterodimer as a search model (PDB code, 2wyb; 34% amino-acid sequence identity)^36^. The C_10_ complex crystallized in the *P*1 space group, similar to that of ligand-free MacQ, while the PAA complex crystallized in the *P*2_1_ space group. The structure of the PAA complex was solved by the molecular replacement method using the ligand-free MacQ as a search model.

**Figure 3:**
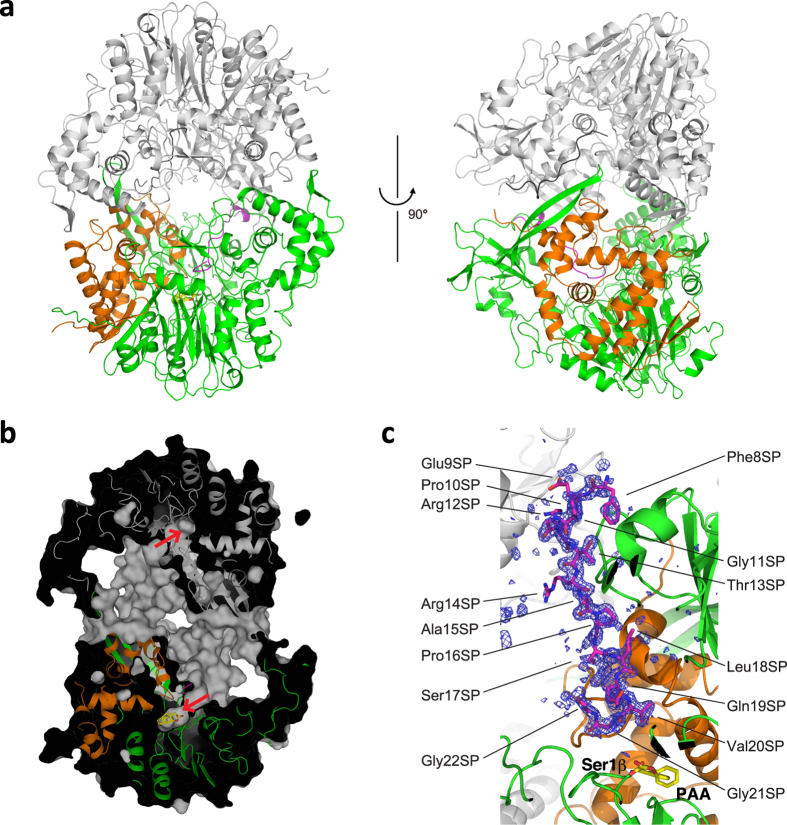
Capsule-shaped assembly of MacQ. (**a**) Ribbon diagram of molecular structure of MacQ formed face-to-face by two heterodimers shown in Fig. 2b. One heterodimer is shown as the same colour scheme to that in Fig. 2b, and the other heterodimer is shown in gray. Two perpendicular views are shown. (**b**) Cross section of the molecule in surface representation showing the interior space of the capsule-like molecule of MacQ and the substrate-binding pocket indicated by the red arrow. The bound PAA is also shown as a yellow stick model. (**c**) *m*Fo-*D*Fc omit map for the observed SP region contoured at 2.6σ level. The final refined model of the SP is shown as stick model and the residues are labelled. The bound PAA and the Ser1β are also shown in stick model. As shown here, the model for the residues Gly1SP-Ala7SP and Glu23SP-Gly27SP are not built due to the poor electron density.

The MacQ structure clearly revealed a typical Ntn-hydrolase fold comprised of a four-layered α-β-β-α motif^45^ mainly composed of two polypeptide chains, α- and β-chains, generated by self-cleaving activity. The N-terminal nucleophile is Ser1β, which corresponds to Ser234 in the precursor sequence (Fig. 2). The DALI structure similarity search^46^ demonstrated the closest structural homology of MacQ to PvdQ [PDB codes, 3L94 (α-chain)^47^/2WYC (β-chain)^36^; Z score, 21.9/49.7; rmsd, 1.2/2.2 Å for 163/546 Cα atoms; sequence identity for the fit region, 38%/35%]. However, two novel findings in the structure of MacQ compared to the structures of PvdQ and other related enzymes were evident. First, electron density was observed for the SP lying in the vicinity of the active-site pocket (Figs 2b and 3c). This was quite unexpected, because the SP was not found in any previously reported structures of penicillin G/cephalosporin*/*AHL-acylases and is believed to fall away in the course of the autoproteolytic maturation. The SP atoms were refined with full occupancy, while the *B*-factor values of the SP were relatively high compared to those of α- and β-chain, indicating increased conformational heterogeneity or a lower occupancy of the SP as compared to the α- and β-chains. Second, the crystal structure analysis revealed that MacQ forms a high oligomeric quaternary structure. The two heterodimers consisting of the α- and β-chain are arranged face-to-face, resulting in the formation of an approximately 170-kDa capsule-shaped molecule (dimer of heterodimer) containing two SP chains (Fig. 3). The unit-cell of the *P*1 lattice contains two capsule-shaped molecules, which are identical and can be superimposed with a root mean squared (RMS) difference of 0.47 Å for 1512 Cα atoms. The asymmetric unit of the *P*2_1_ lattice also contains two PAA-bound molecules, which superimposed well on those of ligand-free and C_10_ complexes of MacQ in the *P*1 lattice, with an RMS difference of ~1.0 Å for 1505 Cα atoms. These results suggest that the lattice forces and the inter-molecular crystal contacts do not affect the overall oligomeric structure of MacQ. Analytical gel filtration also showed that the MacQ predominantly exists as a dimer of heterodimer in solution, although a relatively small amount of heterodimer was also observed (Supplementary Fig. S1).

### Active-site structure and presumed substrate-binding mechanism

To gain insights into the substrate-binding mechanism of MacQ, we attempted to determine the structures of MacQ in complex with substrates (C_10_-HSL and penicillin G). In accordance with a previously reported procedure^36^, the crystals were soaked in saturated substrate solution with a lower pH value (pH 5.5) before X-ray diffraction. The reaction product, C_10_/PAA, was successfully trapped at the hydrophobic active-site pocket, while the electron density for the hydrolyzed counterpart, HSL/6-aminopenicillanic acid (6-APA), was not observed. The results suggest that the more hydrophilic HSL/6-APA diffused into the solvent immediately after the enzymatic amidohydrolyzing reaction.

**Figure 4:**
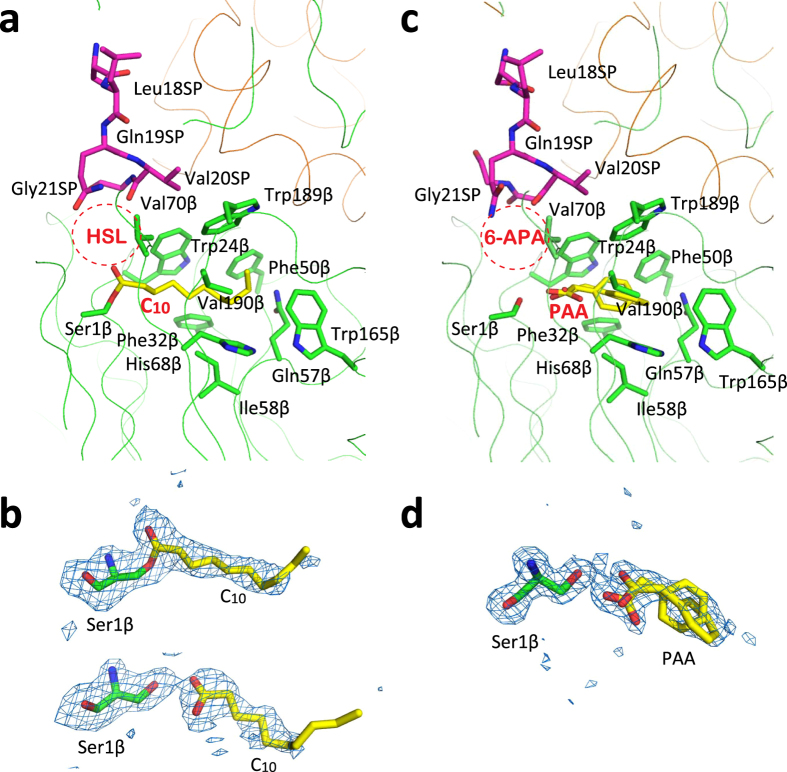
Active site structure of MacQ. The colour scheme is similar to that in Fig. 2b. (**a**) Bound C_10_ fatty acid covalently linked to the Ser1β. The side-chains of nearby residues created the hydrophobic C_10_-binding pocket are also shown and labelled. The expected binding position of HSL moiety is roughly indicated as a red dotted circle. The residues of the SP near the active site are also shown as stick models and labelled. (**b**) *m*Fo-*D*Fc omit map for the bound C_10_ and the Ser1β contoured at 2.5σ level. Two binding states of C_10_ were observed: the covalently linked C_10_ to Oγ of Ser1β (upper) and the noncovalently bound C_10_ (lower). (**c**) Bound PAA. The expected binding position of 6-APA moiety is roughly indicated as a red dotted circle. (**d**) *m*Fo-*D*Fc omit map for the bound PAA and the Ser1β contoured at 2.5σ level. Two PAA molecules modelled with slightly different positions and conformations fit well onto the observed electron density map.

The MacQ homodimer contains two active sites. The C_10_ was found in a different binding state in each active-site pocket. In one active site, the carboxyl group of C_10_ hydrogen-bonded with the side-chain hydroxyl and amino group of the catalytic residue Ser1β, while in the other active site the hydroxyl oxygen (Oγ) of Ser1β was covalently linked to the carbonyl carbon of the C_10_ molecule (Fig. 4a and b). These distinct binding states have also been observed in the structural analyses of PvdQ in complex with dodecanoic acid^36^. Despite the different binding states of C_10_ at the active site pocket, the two heterodimers superimposed well with an RMS difference of ~0.3 Å for 756 Cα atoms. No side-chain conformational changes were detected around the active site, and therefore the factors that cause the different binding modes of the substrate in the different active sites remain unclear. In contrast, PAA was bound in the same state in each active-site pocket without covalent bonds. The atomic models of PAA in two different conformations fit well to the observed electron density and were refined with reasonable *B*-factor values in all active sites (Fig. 4c and d). The C_10_/PAA was accommodated in the highly hydrophobic active-site pocket created by the side-chains of Trp24β, Phe32β, Phe50β, Gln57β, Ile58β, His68β, Val70β, Trp165β, Trp189β, and Val190β (Fig. 4a and c). Amino-acid sequence alignments of MacQ, AhlM, and PvdQ showed that these residues are not completely identical, but are highly conserved (Supplementary Fig. S2). Interestingly, although the induced-fit mechanism for the binding of dodecanoic acid was reported in the structural studies of PvdQ^36^, the active-site of MacQ was determined to be motionless and the side-chain conformations of all the aforementioned residues superimposed well among ligand-free, C_10_ complex, and PAA complex structures, with a constant pocket volume of approximately 170 Å^3^. These observations might partially account for the broad substrate specificity of MacQ.

**Figure 5:**
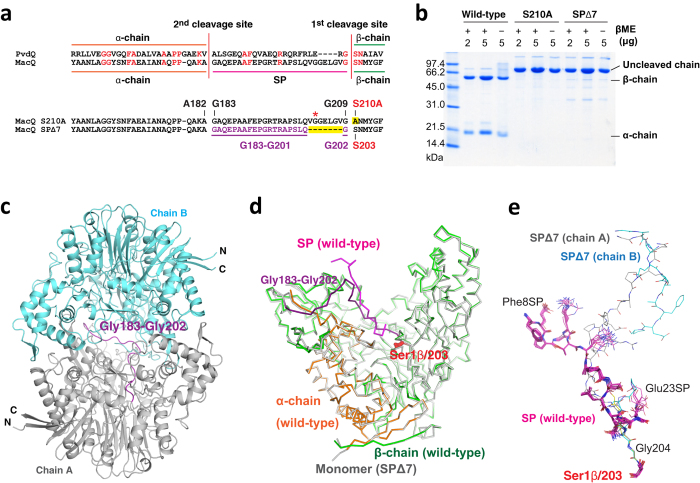
Mutational analyses of MacQ. (**a**) Cleavage site of MacQ and PvdQ and the amino acid sequence of S210A and SPΔ7 mutants around SP region. Deletion or substitutions are highlighted in yellow. The Gly21SP (Gly203 in the uncleaved form), the nearest SP residue to the active site, is indicated by the red asterisk. The seven-residue deletion is the minimum requirements to delete the Gly21SP (Gly203) in the cleaved form of MacQ with keeping the cleavage sequence Gly209-Ser210. (**b**) SDS-PAGE analysis of wild-type MacQ and mutants S210A and SPΔ7 under reducing (β-mercaptoethanol (βME) + ) and non-reducing (βME −) conditions. The results clearly show that both S210A and SPΔ7 mutants were not cleaved. (**c**) Overall structure of MacQ SPΔ7. The structure shows the same capsule-like molecule as that of wild-type MacQ, but consists of two uncleaved monomers. The residues 183–202, which correspond to the SP of the wild-type enzyme, are coloured in dark magenta. (**d**) Structure superimposition of wild-type MacQ heterodimer and SPΔ7 monomer. The α-chain, SP, and β-chain of wild-type MacQ are coloured in orange, magenta, and green, respectively. The SPΔ7 is coloured in gray, while the Gly183-Gly202 is coloured in dark magenta, as shown in (a). The conformation of the SP region of wild-type MacQ and the corresponding Gly183-Gly202 of SPΔ7 is completely different. (**e**) Structure superimposition of the SP/Ser1β of wild-type MacQ in ligand-free and C_10_/PAA complex and the Gly183-Ser203 of the SPΔ7. A total of 12 chains for wild-type MacQ and 4 chains for SPΔ7 are shown. The SPΔ7 residues of 193–197 for chain B, 178–200 for chain C and 179–200 for chain D could not be built due to poor electron density. The carbon atoms are coloured in magenta for the SP of wild-type enzyme, and in gray for chain A, cyan for chain B, yellow for chain C, and light blue for chain D of the SPΔ7.

The remaining SP in the hollow surface of the molecule is probably serendipitous, but we presume that the SP could be involved in MacQ function for the following reasons. First, the SP is observed in all heterodimeric units of MacQ in the same conformation, both in the presence and absence of ligands (Fig. 5e). Second, the residues from Gln19SP to Gly22SP form a type-II β-turn structure together with ordered solvent molecules, and the main-chain atoms that form a peptide linkage between the Val20SP and Gly21SP lie at the entrance of the hydrophobic pocket, where the residues can interact with the HSL/6-APA moiety of the bound substrate. The distance between Oγ of the Ser1β and the peptide-bond atoms of Val20SP-Gly21SP is ~7 Å. It is considered that the loose binding of C_10_/PAA without an induced-fit mechanism might be compensated for by the presumed interactions between SP and HSL/6-APA. To investigate whether the SP is important for enzymatic function of MacQ, we created an SP-shortened mutant and examined its enzymatic activity level.

### Spacer-shortened mutant MacQ is an enzymatically active single polypeptide

The self-cleaving maturation mechanism has been extensively studied using cephalosporin acylase (CA)^37–39, 48^. Various SP-shortened mutants of CA were created^48^. They retained self-cleaving activity similar to wild-type CA. On the basis of the previous reports regarding CA, we created an SP-shortened MacQ by deleting seven residues from Val20SP to Val26SP (from Val203 to Val209; Fig. 5a). We expected that the SP-shortened mutant, termed SPΔ7, would provide important clues for the enzymatic function of the C-terminal region of the SP. The recombinant SPΔ7 was expressed and purified by the same procedure as the wild-type enzyme. Unexpectedly, SDS-PAGE analysis showed that the purified SPΔ7 was uncleaved and was present as a single polypeptide chain with a subunit weight of approximately 80 kDa (Figs. 5a and b), suggesting that the seven-residue deletion of the SP inactivated the self-cleaving maturation, despite the fact that SPΔ7 possesses the catalytic serine (Ser1β in the cleaved form). We also investigated whether SPΔ7 retains AHL/penicillin G acylase activity. Interestingly, SPΔ7 exhibited unambiguous degrading activity against almost all of the substrates tested for wild-type MacQ (Table 1, Supplementary Figs S3 and S4)^3^. These results indicate that the self-cleaving maturation is not a requisite process for the enzymatic activation of MacQ. In light of this finding, we were interested in determining the crystal structure of SPΔ7 to understand how the uncleaved polypeptide chains fold and form the active site.

**Table 1:** Summary of AHLs/penicillin G degrading activity of wild-type MacQ and the mutants, SPΔ7 and S210A.

	AHLs ((O)C_x_-HSL)^*^									PenG^#^	SC^‡^
	C_6_	C_8_	C_10_	C_12_	OC_6_	OC_8_	OC_10_	OC_12_	OC_14_		
Wild-type	+	+	+	+	+	+	+	+	+	+	+
SPΔ7	+	+	+	+	−	+	+	+	+	+	−
S210A	−	+	+	+	−	−	+	+	+	−	−

^*^Abbreviations of AHLs are: C_6_-HSL, *N*-hexanoyl-l-homoserine lactone; C_8_-HSL, *N*-octanoyl-l-homoserine lactone; C_10_-HSL, *N*-decanoyl-l-homoserine lactone; C_12_-HSL, *N*-dodecanoyl-l-homoserine lactone; OC_6_-HSL, *N*-(3-oxo-hexanoyl)-l-homoserine lactone; OC_8_-HSL, *N*-(3-oxo-octanoyl)-l-homoserine lactone; OC_10_-HSL, *N*-(3-oxo-decanoyl)-l-homoserine lactone; OC_12_-HSL, *N*-(3-oxo-dodecanoyl)-l-homoserine lactone; OC_14_-HSL, and *N*-(3-oxo-tetradecanoyl)-l-homoserine lactone.

^#^PenG, penicillin G.

^‡^SC, self-cleaving (autoproteolytic) activity.

### Structure analysis of MacQ SPΔ7 mutant

We successfully determined the crystal structure of MacQ SPΔ7 in ligand-free form. SPΔ7 crystals were obtained only using a different crystallization solution containing tacsimate buffer (pH 6.0), but the crystals displayed unit-cell parameters with an identical space group similar to those of the PAA complex of the wild-type MacQ. Unfortunately, attempts to determine the structure of ligand-bound SPΔ7 were unsuccessful because of the low reproducibility and fragility of SPΔ7 crystals. The asymmetric unit contains two capsule-like molecules that form the same structure as the wild-type MacQ. However, each molecule was determined to be a homodimer composed of two uncleaved SPΔ7 polypeptides (Fig. 5b,c, and d). The overall structure of SPΔ7 superimposed very well on that of the wild-type enzyme without its SP region (Fig. 5d and e). We could build the continuous atomic model for chain A (Supplementary Fig. S5), while several residues corresponding to the SP region of the wild-type enzyme were disordered for the chain B, and most of the residues of the SP region (residue 179–200) were disordered for chains C and D. The conformation of the SP region of the SPΔ7 (residue 183–202) was found to be completely different from that of the SP in the wild-type enzyme (Fig. 5e).

The structure of SPΔ7 indicates a possible mechanism of the autoproteolytic cleavage between Gly202 and Ser203 (Supplementary Fig. S6). It is likely that the water molecule that hydrogen-bonds with the main-chain O of Gln201 and His225 (Wat X in Supplementary Fig. S6) forms the pseudo-tetrahedral geometry and deprotonates the side-chain hydroxyl of Ser203. The activated hydroxyl of Ser203 then carries out the nucleophilic attack on the main-chain C of the Gly202. The oxyanionic intermediate is likely stabilized by the main-chain amide of Val272, thereby lowering the activation energy for the peptide bond cleavage. In the SPΔ7 structure, the ~3.4 Å distance between the main-chain O of Gly202 and the main-chain amide of Val272 would be too distant to form a hydrogen bond. The seven-residue deletion may affect the main-chain conformation of the Gly202-Ser203, leading to the destabilization of the intermediate structure. The SPΔ7 structure also revealed that the side-chain N of Asn480 approaches the main-chain O of Gly202 in a direction nearly vertical to the peptide plane. The conformation of the “Gly-Ser” in the wild-type MacQ precursor might have a more favourable inter-atomic geometry to access the oxyanion hole created by the main-chain N of Val272 and possibly the side-chain N of Asn480 (Supplementary Fig. S6).

**Figure 6:**
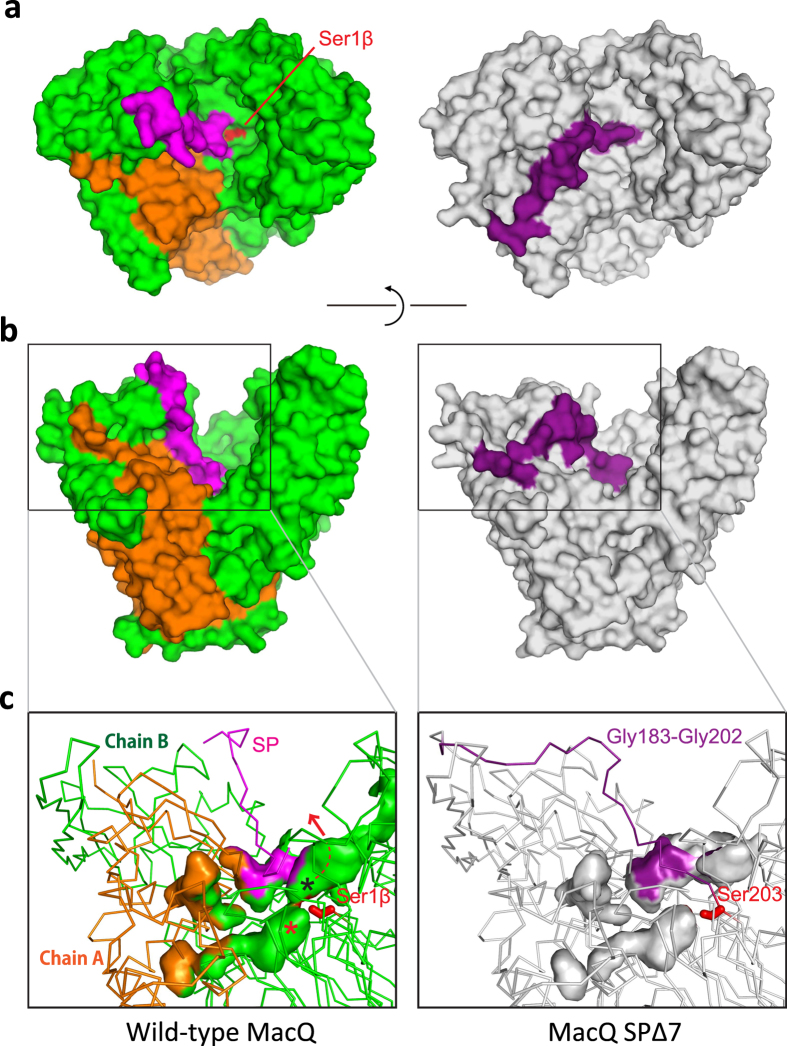
Comparison of molecular surface and active site cavities of wild-type MacQ and SPΔ7 mutant. (**a**) Top view and (**b**) side view molecular surface representation of heterodimeric wild-type MacQ and monomeric (uncleaved) SPΔ7. The colour scheme corresponds to Fig. 5d. The Ser1β of wild-type MacQ is located at the depth of the active-site cleft and is accessible from the solvent, while the corresponding Ser203 in SPΔ7 is buried inside due to the presence of uncleaved chain. (**c**) Active site cavities of the heterodimeric wild-type MacQ and monomeric (uncleaved) SPΔ7. The Ser1β/203 is represented as a stick model and coloured in red. C_10_/PAA binding pocket and the putative HSL/6-APA binding position are indicated by asterisk in red and black, respectively. The red arrow represents the putative releasing route of the HSL/6-APA from the pocket in the vicinity of Ser1β. The corresponding pocket is not formed in the structure of SPΔ7 due to the presence of uncleaved polypeptide chain.

Because the uncleaved SPΔ7 is enzymatically active for both AHLs and penicillin G, the question arises as to how the SPΔ7 recognizes the substrate at the active site. The hydrophobic active-site pocket where the C_10_/PAA is bound in the wild-type enzyme is also present in the structure of SPΔ7. However, the pocket is insulated from the solvent because of the presence of the uncleaved polypeptide chain (residues Leu201-Ser203) (Fig. 6). Thus, it is likely that certain local conformational changes are required for the binding of the substrate and for catalytic reaction. To check whether the Ser210 (corresponding to Ser203 of SPΔ7 or Ser1β of wild-type enzyme) is really essential for autoproteolytic activation and AHL/penicillin G-degrading function^31, 49, 50^, we created a S210A mutant and examined its enzymatic activity.

### Uncleaved S210A mutant is also active for AHLs

The recombinant S210A MacQ mutant was expressed and purified by the same procedure as the wild-type enzyme and SPΔ7. SDS-PAGE analysis of the purified sample showed that the S210A mutant is also present as an uncleaved form, similar to the SPΔ7 mutant (Fig. 5b). Ntn is also essential for self-cleaving maturation as well as enzymatic activity^39–43^. Thus, it is reasonable that the S210A mutant was uncleaved. Next, we assessed whether the S210A mutant is enzymatically active. The mutant could not degrade penicillin G, but was enzymatically active for various AHLs (Table 1, Supplementary Figs S3 and S4). Although the mechanisms of substrate binding and catalytic reaction of the S210A mutant remain unclear, it is evident that neither self-cleaving maturation nor Ser210 are essential for AHL acylase activity of MacQ, except for the C_6_-HSL, OC_6_-HSL and OC_8_-HSL short-chain AHLs.

## Conclusions

MacQ is an enzyme belonging to the Ntn hydrolase superfamily. It catalyzes the amidohydrolase reaction for various ranges of AHLs and for β-lactam antibiotics. We determined the crystal structures of MacQ in ligand-free form and complexed with the C_10_ and PAA reaction products. MacQ forms a previously unreported 170-kDa capsule-like molecule consisting of a self-cleaved α-chain, SP, and β-chain. The SP showed increased conformational heterogeneity compared to the α- and β-chain, while the peptide-bond atoms of Val20SP-Gly21SP, which show the relatively low *B*-factor values in the structure of the SP, lie in the vicinity of the active-site pocket, likely accessible to the hydrophilic HSL/6-APA moiety of the bound substrate. In addition, both SP-shortened SPΔ7 and S210A mutants of MacQ result in the uncleaved, immature form of the protein, but retain amidohydrolyzing activity against AHLs, except for the short-chain AHLs, C_6_-HSL and OC_6_-HSL. These results suggest that MacQ is a structurally and enzymatically unique protein that does not conform to the common theory of autoproteolytic activation of Ntn-hydrolases and exemplifies the versatility of highly diversified Ntn hydrolase superfamily^45^. Future work will aim at investigating the underlying enzyme mechanism of MacQ by further mutational and structural analyses.

## Methods

### Recombinant protein expression and purification

The expression construct for wild-type MacQ was previously described^3^. The SPΔ7 and S210A mutants were created by the inverse PCR method^51^. Overexpression and purification of recombinant MacQ was conducted as previously described^3^. Briefly, overexpression of MacQ and its mutants with His-tags at both the N- and C-termini were induced by addition of 0.1 mM isopropyl-β-d-thiogalactopyranoside (IPTG) for 18 hr at 18 °C, using *E. coli* strain Origami^TM^ 2 (DE3) (Novagen). After cell disruption by sonication, the supernatant was applied to a Ni affinity chromatography column (HIS-SELECT; Sigma-Aldrich), and the His-tagged sample was eluted according to the manufacturer’s instruction. The molecular weights of the subunit components of purified MacQ were confirmed with a Voyager DE Pro matrix-assisted laser desorption ionization time-of-flight (MALDI-TOF) mass spectrometer (Applied Biosystems). The α-chain of MacQ was separated by SDS-PAGE and transferred onto polyvinylidine difluoride (PVDF) membranes. The Coomassie Blue R250-stained bands on PVDF membranes were excised and applied to the N-terminal sequence analyses (Procise 492HT peptide sequencer; Applied Biosystems). The protein concentration was determined using Bradford protein assay kit (BioRad) with bovine serum albumin as a standard. The purified sample was dialyzed against the buffer consisting of 20 mM Tris-HCl pH 7.5, 50 mM NaCl, and 1 mM dithiothreitol (DTT) and was concentrated to 8 mg/mL for crystallization, using a centrifugal filter (molecular weight cut-off, 100 kDa; Millipore).

### Analytical gel-filtration chromatography

The purified wild-type MacQ (cleaved form) and the S210A mutant (uncleaved form) were loaded on a Superose 6 10/300 GL column (GE Healthcare) equilibrated with 50 mM sodium phosphate pH 8.0 and 200 mM NaCl, at a flow rate of 0.4 mL/min. Ferritin (440 kDa), catalase (232 kDa), aldolase (158 kDa), ovalbumin (43 kDa) and ribonuclease A (13.7 kDa) were used as molecular-weight standards to calibrate the column. The molecular weight standard proteins were obtained from GE Healthcare.

### Enzyme assay

The activities of MacQ and the generated mutants against various ranges of AHLs were examined by an AHL-inactivation assay using the green fluorescence protein (GFP)-based biosensor strains *E. coli* MT102 (harbouring plasmid pJBA132) and *P. putida* F117 (harbouring plasmid pKR-C12)^52, 53^, as previously described^3^. The amidohydrolyzing activity against penicillin G was examined by analyzing the reaction products by gas chromatography-mass spectrometry (GC-MS), as previously described^3^.

### Crystallization

All crystallization screenings were carried out at 20 °C by the vapor-diffusion technique. Initial crystallization screening was performed using commercially available sparse matrix screening kits (Hampton Research and Emerald BioStructures), by the sitting-drop vapor-diffusion method in 96-well plates. Drops were formed by mixing 0.5 μL of sample and an equal volume of reservoir solution, and were equilibrated against 70 μL reservoir solution. Subsequent optimization of the hit conditions was performed using hanging-drop vapor-diffusion using 24-well plates. The crystals of wild-type MacQ were obtained using the reservoir solution consisting of 100 mM Tris-HCl pH 7.5–8.5, 0.1–0.2 M calcium acetate, and 14–18% polyethylene glycol (PEG) 3350. The SPΔ7 mutant was also crystallized using the reservoir solution consisting of 8% tacsimate pH 6.0 and 16% PEG 3350.

### Structure determination and model refinement

Prior to X-ray diffraction, crystals were soaked into the cryoprotectant solution consisting of crystallization mother liquor supplemented with 20% glycerol, and were flash-cooled with a nitrogen gas stream at 100 K. For the data collection of the ligand complex, the crystals were briefly soaked in a freshly prepared solution consisting of 100 mM bis-Tris pH 5.5, 0.2 M calcium acetate, 18% PEG 3350, 20% glycerol, and saturated substrate (C_10_-HSL/penicillin G). X-ray diffraction data were collected with synchrotron radiation at the Photon Factory (Tsukuba, Japan). The raw data were processed using the HKL2000 program package^54^. The crystals of ligand-free and C_10_-bound MacQ belong to the space group *P*1 with unit-cell parameters approximately *a = *86, *b* = 90, *c* = 123 Å, *α* = 103, *β* = 105, and *γ* = 106°. The crystals of wild-type MacQ with PAA complex and the SPΔ7 mutant belong to the space group *P*2_1_ with unit-cell parameters approximately *a = *102, *b* = 139, *c* = 122 Å, *α* = 90, *β* = 111 and *γ* = 90°. The structure of ligand-free MacQ was determined by the molecular replacement method with the program PHASER^55^ using the PvdQ heterodimer (PDB code, 2WYB) as a search model. After density modification by the program DM^56^, manual model building and iterative model fitting were performed with the graphics program COOT^57^. The structures of PAA-bound MacQ and the SPΔ7 mutant in space group *P*2_1_ were determined by molecular replacement method using the ligand-free MacQ as a search model. Model refinement was performed using the program REFMAC5^58^. The stereochemical quality of the final refined model was performed using the program PROCHECK^59^. Molecular drawings were prepared using the program PyMOL (http://www.pymol.org/). Crystallographic parameters and refinement statistics are summarized in Supplementary Table S1.

### Data availability statements

The protein structures reported in this study have been deposited in the RCSB Protein Data Bank (http://www.rcsb.org) under accession codes 4YF9, 4YFA, 4YFB and 5C9I. Other data generated during and/or analyzed during the current study are available from the corresponding author on reasonable request.

## Electronic supplementary material

Supplementary Information
