# Supplementary material for: Bifunctional quorum-quenching and antibiotic-acylase MacQ forms a 170-kDa capsule-shaped molecule containing spacer polypeptides
Source: Sci Rep. 2017 Aug 21;7:8946. doi: 10.1038/s41598-017-09399-4 (PMC5566955; doi:10.1038/s41598-017-09399-4)
Supplement: Supplementary file 1 — Supplementary Information [file 41598_2017_9399_MOESM1_ESM.pdf]

Supplementary information:

Bifunctional quorum-quenching and antibiotic-acylase MacQ forms a 170-kDa capsule-shaped molecule containing spacer polypeptides

Yoshiaki Yasutake[1]*, Hiroyuki Kusada[2,3], Teppei Ebuchi[2,3], Satoshi Hanada[2], Yoichi Kamagata[2], Tomohiro Tamura[1], Nobutada Kimura[2,3]

[1]Bioproduction Research Institute, National Institute of Advanced Industrial Science and Technology (AIST), 2-17-2-1 Tsukisamu-Higashi, Toyohira, Sapporo 062-8517, Japan

[2]Bioproduction Research Institute, National Institute of Advanced Industrial Science and Technology (AIST), 1-1-1 Higashi, Tsukuba, Ibaraki 305-8566, Japan

[3]Graduate School of Life and Environmental Sciences, University of Tsukuba, 1-1-1 Ten-nodai, Tsukuba, Ibaraki 305-8572, Japan

*Correspondence should be addressed to Y.Y. E-mail, y-yasutake@aist.go.jp.

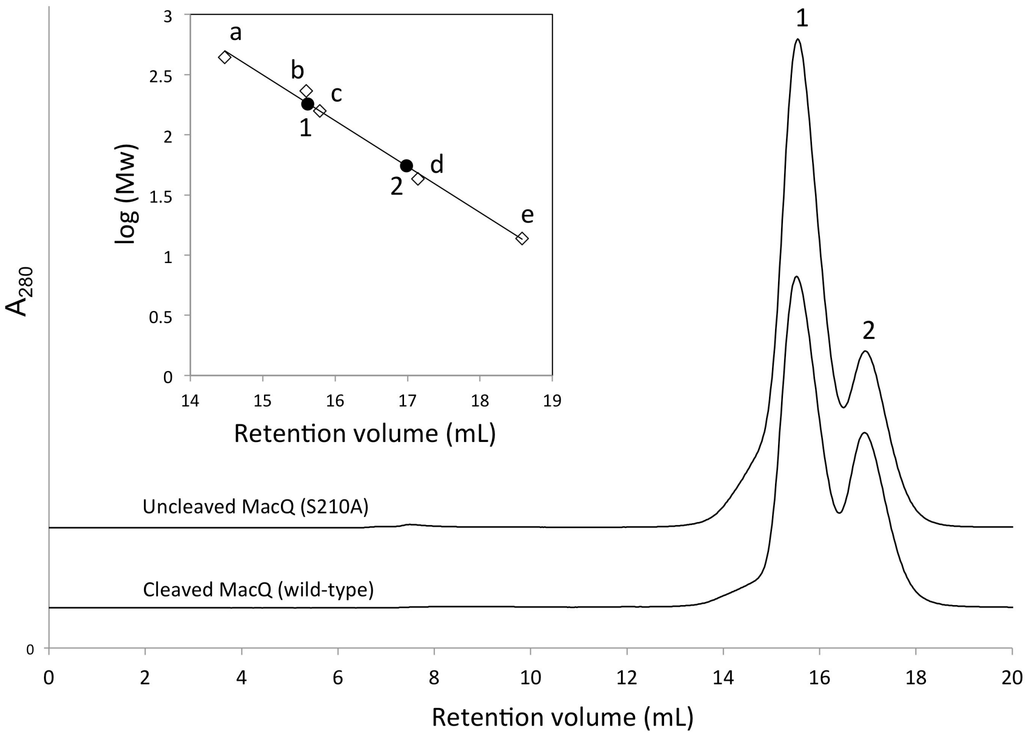

Supplementary Figure S1. Estimation of molecular weight of wild-type MacQ (cleaved form) and the S210A mutant (uncleaved form) in solution state by analytical gel-filtration. The column was calibrated with ferritin (a, 440 kDa), catalase (b, 232 kDa), aldolase (c, 158 kDa), ovalbumin (d, 43 kDa) and ribonuclease A (e, 13.7 kDa). The molecular weight corresponding to the elution peak 1 and 2 is estimated to be approximately 200 kDa and 58 kDa, respectively.

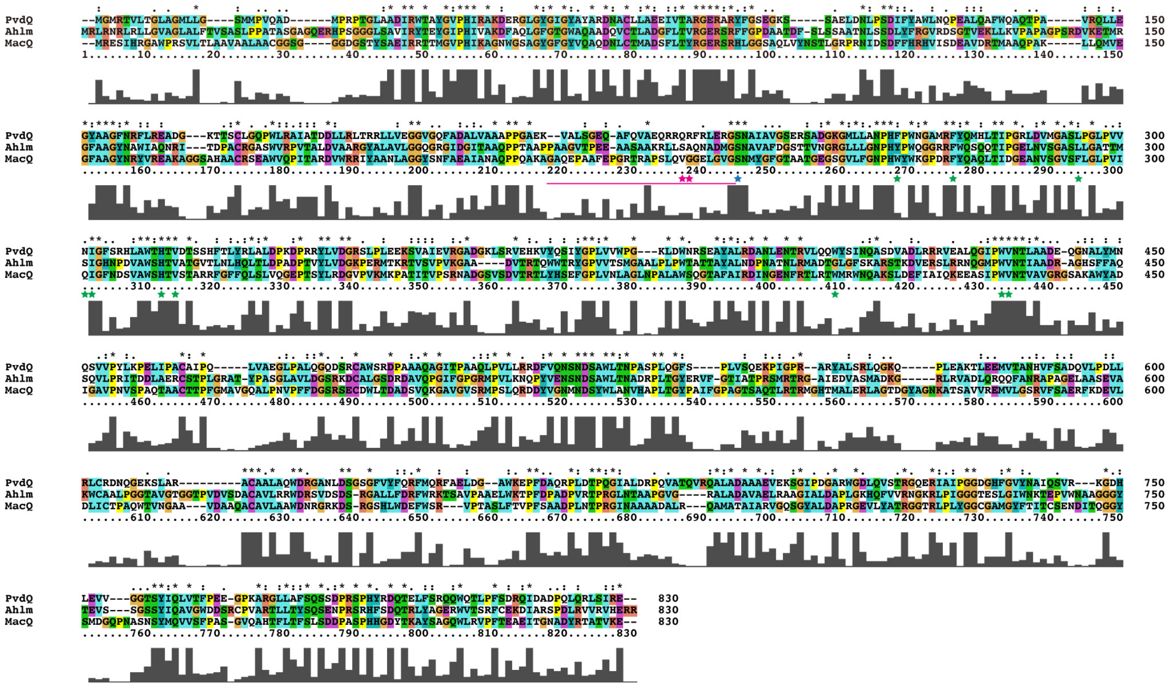

Supplementary Figure S2. Multiple sequence alignment between MacQ, AhlM from *Streptomyces* sp. M664, and PvdQ from *Pseudomonas aeruginosa* PAO1. AhlM is known as an enzyme that is capable of degrading both AHLs and penicillin G. PvdQ is the most structurally homologous enzyme to the MacQ, as revealed by DALI structure similarity search. The sequence alignment was performed and presented using the program Clustal X 2.0 with default parameters. The SP is depicted by the line in magenta. The residues creating the hydrophobic C_{10}/PAA binding pocket shown in Fig. 3 are marked by green stars. The residues of SP in the vicinity of active site (Val20SP and Gly21SP) are marked by magenta stars. The catalytic Ser1β is marked by a blue star.

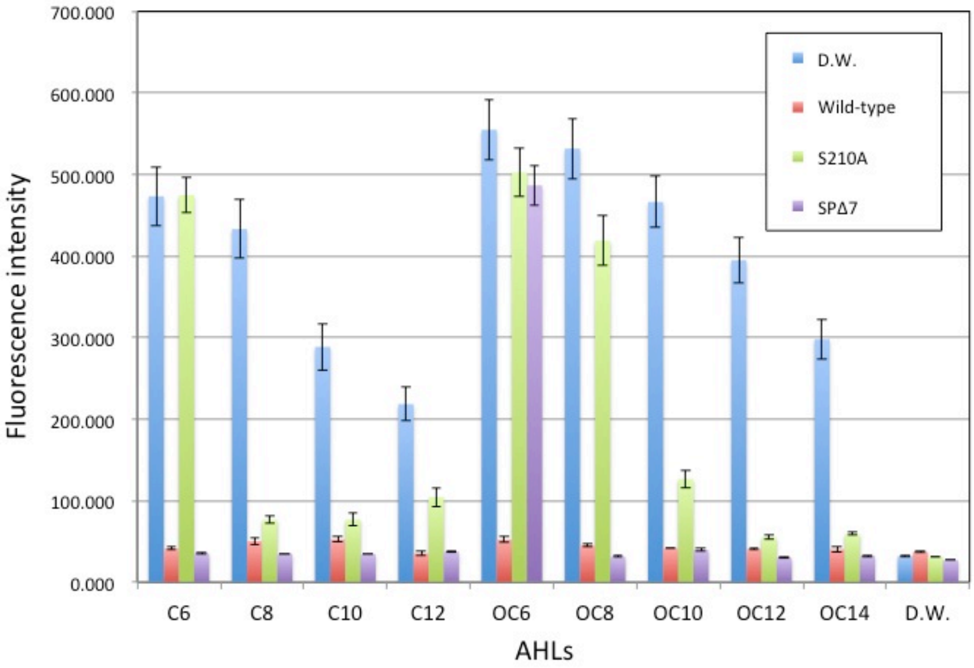

Supplementary Figure S3. The results of the enzyme bioassay for AHL-degrading activity of wild-type MacQ, S210A and SPΔ7. A total of nine AHLs were tested (see Table 1). Mean fluorescence intensity ± SD are shown, as calculated from triplicate experiments. D.W., distilled water, added in place of enzyme/AHLs. The results are also summarized in Table 1.

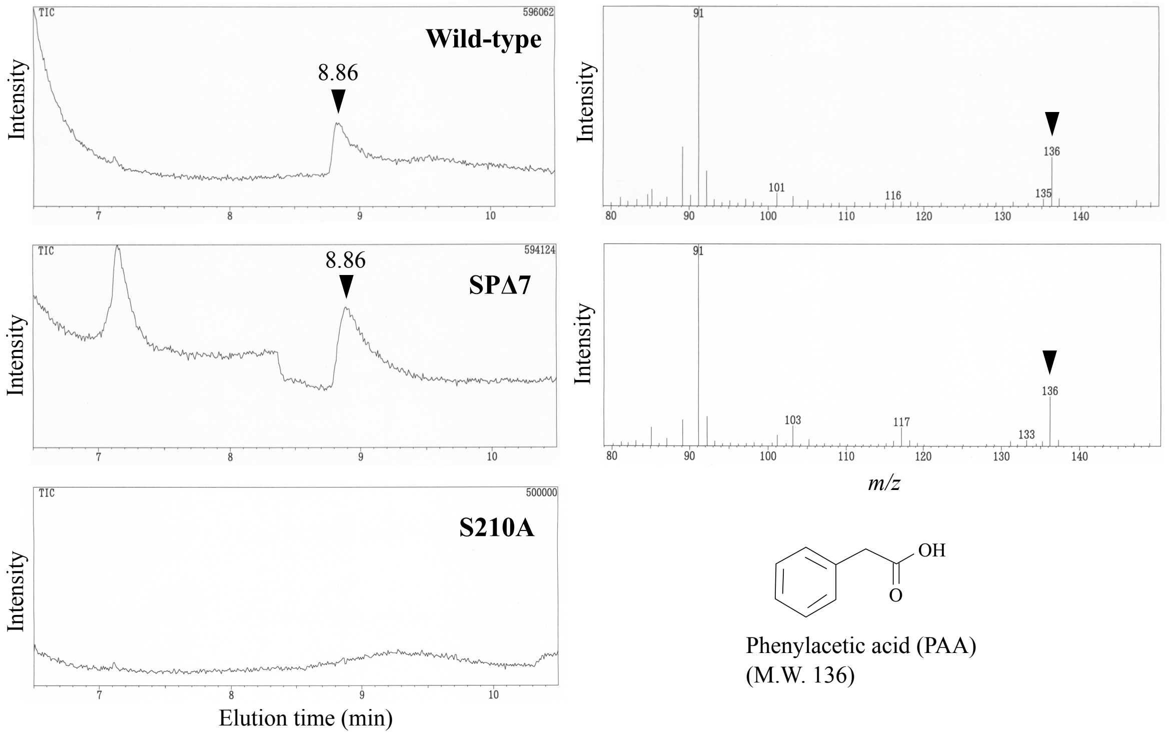

Supplementary Figure S4. The results of GC-MS analysis of penicillin G metabolite degraded by wild-type MacQ, S210A, and SPΔ7. The results clearly showed that wild-type MacQ and SPΔ7 have aminohydrolase activity against penicillin G.

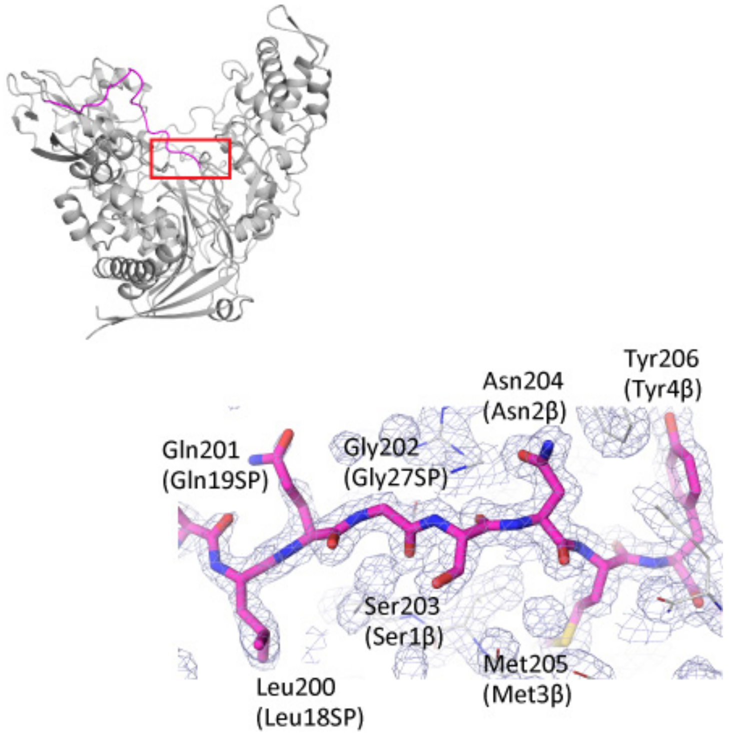

Supplementary Figure S5. Fo-Fc omit map for the residues Leu200-Tyr206 of SPΔ7 that correspond to Leu18SP-Tyr4β of wild-type MacQ. The map is shown contoured at the 2.6σ level, showing that the continuous electron density between Gly202 and Ser203.

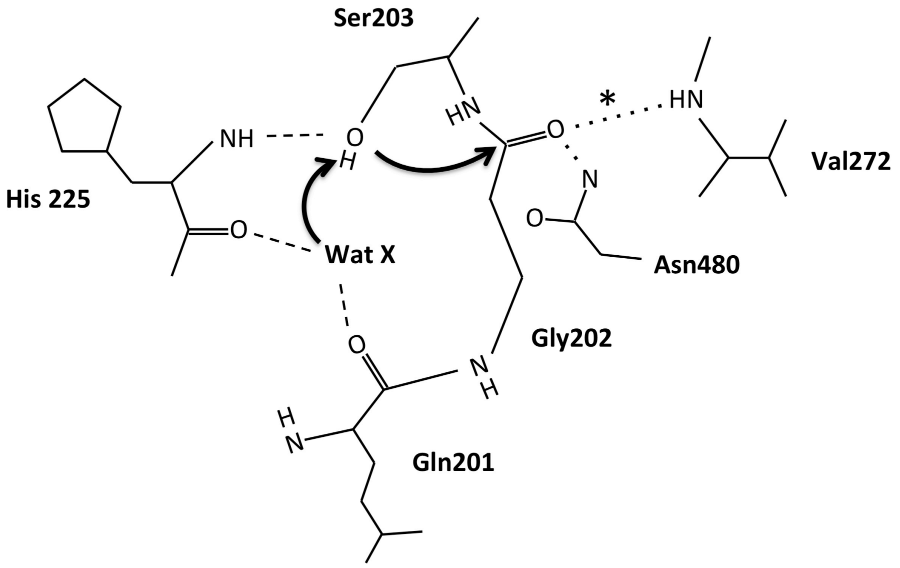

Supplementary Figure S6. Proposed mechanism of the autoproteolytic cleavage between Gly202 and Ser203. The residue numbering is based on the structure of SPΔ7. In the crystal structure of SPΔ7, Wat X corresponds to Wat1012 (chain A), Wat929 (chain B), Wat843 (chain C) and Wat898 (chain D). The inter-atomic distance between main-chain O of Gly202 and the main-chain N of Val272, indicated by asterisk, is ~3.4 Å. The side-chain N of Asn480 is also located close to the main-chain O of Gly202 but in a direction nearly vertical to the peptide plane. The more favorable inter-atomic geometry might be present in the wild-type MacQ precursor to stabilize the oxyanion intermediate and facilitate the peptide bond cleavage.

Supplementary Table S1. Crystallographic data and refinement statistics.

	Wild-type	Wild-type	Wild-type	SPΔ7 mutant
Bound ligand	-	Decanoic acid (C_{10})	Phenyl acetic acid (PAA)	-
PDB code	4YF9	4YFA	4YFB	5C9I
Data collection				
Beamline	BL-5A	AR-NE3A	AR-NE3A	BL-17A
Wavelength (Å)	1.0000	1.0000	1.0000	0.9800
Space group	$P1$	$P1$	$P2_1$	$P2_1$
Unit-cell parameters a, b, c (Å), α, β, γ (°)	85.6, 90.1, 123.2, 103.5, 105.0, 106.0	85.4, 89.9, 122.7, 103.3, 104.8, 106.1	102.5, 139.0, 122.0, 90.0, 111.1, 90.0	102.7, 137.8, 121.5, 90.0, 111.5, 90.0
Resolution range (Å)	50–2.60 (2.67–2.60)	50–2.20 (2.24–2.20)	50–1.75 (1.78–1.75)	50–1.80 (1.83–1.80)
Unique reflections	98,361	159,207	319,483	290,370
Redundancy	3.9 (3.9)	3.9 (3.9)	3.8 (3.7)	3.8 (3.7)
Completeness (%)	98.9 (98.2)	98.3 (97.3)	99.9 (99.7)	100.0 (99.9)
$I/\sigma(I)$	15.0 (2.8)	23.4 (2.3)	16.3 (3.2)	12.8 (2.4)
R_{merge}*	0.101 (0.638)	0.083 (0.735)	0.081 (0.539)	0.117 (0.792)
Model refinement				
R_{work}/R_{free}#	0.196/0.241	0.193/0.235	0.161/0.193	0.166/0.206
r.m.s.d. bond lengths (Å)	0.007	0.007	0.021	0.020
r.m.s.d. bond angles (°)	1.10	1.18	1.96	1.88
Total atoms	23,165	23,903	25,503	25,338
Average B-factors (Å^2)				
Overall	42.7	45.9	21.3	21.3
Ligand	-	44.4 (C_{10})	20.4 (PAA)	-
Solvent	27.2	40.1	27.1	26.7
Ramachandran plot				
Favored (%)	89.2	89.2	90.9	89.4
Allowed (%)	10.6	10.6	8.9	10.2
Disallowed (%)‡	0.2	0.2	0.2	0.4

Values in parentheses refer to data in the highest resolution shell.

*$R_{merge} = \Sigma_h \Sigma_i |I_{h,i} - \langle I_h \rangle| / \Sigma_h \Sigma_i I_{h,i}$, where $\langle I_h \rangle$ is the mean intensity of a set of equivalent reflections.

#$R_{work} = \Sigma |F_{obs} - F_{calc}| / \Sigma F_{obs}$ for 95% of the reflection data used in the refinement. F_{obs} and F_{calc} are observed and calculated structure factor amplitudes, respectively. R_{free} is the equivalent of R_{work}, except that it was calculated for a randomly chosen 5% test set excluded from refinement.

‡The residues found in the disallowed regions of the Ramachandran plot have well-defined electron density and their main-chain conformations are reliable.
